# Time in blood glucose range 70 to 180 mg/dL and survival rate in critically ill patients: A retrospective cohort study

**DOI:** 10.1371/journal.pone.0252158

**Published:** 2021-05-27

**Authors:** Hiromu Naraba, Tadahiro Goto, Toru Shirakawa, Tomohiro Sonoo, Naoki Kanda, Hidehiko Nakano, Yuji Takahashi, Hideki Hashimoto, Kensuke Nakamura

**Affiliations:** 1 Department of Emergency and Critical Care Medicine, Hitachi General Hospital, Hitachi, Ibaraki, Japan; 2 TXP Medical Co., Ltd., Bunkyo, Tokyo, Japan; 3 Department of Clinical Epidemiology and Health Economics, School of Public Health, The University of Tokyo, Bunkyo, Tokyo, Japan; 4 Department of Social Medicine, Public Health, Osaka University Graduate School Medicine, Suita, Osaka, Japan; International University of Health and Welfare, School of Medicine, JAPAN

## Abstract

**Background:**

While time in targeted blood glucose range (TIR) 70–140 mg/dL is a known factor associated with mortality in critically ill patients, it remains unclear whether TIR is associated with 28-day mortality under the glycemic control with a less tight target glucose range of 70–180 mg/dL. We aimed to examine whether TIR 70–180 mg/dL was associated with 28-day mortality.

**Methods:**

This is a retrospective cohort study using data from a tertiary care center in Japan collected from January 2016 through October 2019. We included adult patients (aged ≥20 years) admitted to the ICU. We excluded patients 1) with diabetic ketoacidosis patients, 2) discharged within 48 hours, 3) with repeated ICU admissions. We calculated TIR 70–180 mg/dL using the measured blood glucose values (≥3 times per day). The primary outcome was 28-day mortality. We examined the association between TIR and 28-day mortality using a logistic regression and Cox proportional hazard models with a stratification by glycosylated hemoglobin (HbA1c) level of 6.5%. Additionally, we repeated the analyses using the TIR category to assess the optimal TIR. For the sensitivity analysis, we repeated the primary analysis using TIR during the first three days from ICU admission.

**Results:**

Of 1,230 patients, the median age was 72 years, 65% were male, and 250 patients (20%) had HbA1c ≥6.5% on admission. In patients with HbA1c <6.5%, TIR <80% was associated with an increased risk of 28-day mortality, with an adjusted odds ratio (OR) of 1.88 (95%CI: 1.36–2.61). Likewise, when using 10% incremental TIR as a categorical variable, lower TIR was associated with a worse 28-day mortality compared with TIR ≥90% (e.g., adjusted OR of TIR <60%, 3.62 [95%CI 2.36–5.53]). Similar associations were found in the analyses using Cox proportional hazards model and using TIR during the first three days. By contrast, in patients with HbA1c ≥6.5%, there was no consistent association of TIR with 28-day mortality.

**Conclusions:**

We found that lower TIR 70–180 mg/dL was associated with a higher 28-day mortality in critically ill patients with HbA1c <6.5%, whereas there was no consistent association in patients with HbA1c ≥6.5%.

## Introduction

Glycemic control for critically ill patients remains an important issue related to critical care. Although intensive insulin therapy had been recommended [1, 2], a large randomized controlled trial, "NICE-SUGAR study" demonstrated higher mortality in the intensive insulin therapy group (i.e., interventional arm) [3]. Therefore, the current recommendations for glycemic control management in critically ill patients have been changed from "intensive insulin therapy" to "manage with less tight control with the limit of <180 mg/dL." [4–6].

Recently, the importance of time in targeted blood glucose range (TIR) has been emphasized as a prognostic factor for critically ill patients [7–11]. In previous studies, TIR 70–140 mg/dL <80% was associated with a higher mortality in nondiabetic critically ill patients with HbA1c ≤6.5% [9]. However, current national guidelines recommend the more liberal upper target glucose level of 180 mg/dL [12]. Indeed, the *Surviving Sepsis Campaign Guidelines* suggest the upper limit of the glucose level at ≤180 mg/dL [6]. Furthermore, a systematic review of 36 randomized controlled trials and the *American Diabetes Association* also recommend a target glucose range of 140–180 mg/dL for most critically ill patients (grade A) [13]. Despite the clinical consensus on the liberal upper target glucose level of 180 mg/dL, little is known about the association between glycemic control with TIR 70–180 mg/dL and prognosis of critically ill patients. Additionally, it remains unclear whether the optimal TIR for critically ill patients should be maintained at a target upper glucose level of ≤180 mg/dL.

To address the knowledge gap in the literature, we examined the association of TIR 70–180 mg/dL with 28-day mortality among patients admitted to the intensive care unit (ICU). We hypothesized that lower TIR would be associated with a higher risk of 28-day mortality in patients admitted to the ICU.

## Materials and methods

### Study design and setting

This retrospective cohort study used data from the Hitachi General Hospital from 1 January 2016 through 31 October 2019. Hitachi General Hospital, a tertiary care center in Japan, serves an area with approximately three million residents. Annual emergency department visits are about 27,000 encounters. The Hitachi General Hospital has 18-beds ICU and 6-beds cardiac care unit. Of the 18 ICU beds, eight beds have a 2:1 patient-nurse ratio. The remaining ten beds have a 4:1 patient-nurse ratio. The ICU physicians manage all critically ill patients (both intrinsic [e.g., respiratory failure, heart failure, sepsis, stroke, gastrointestinal perforation] and extrinsic [e.g., suffocation, toxicity, burn, trauma] conditions except for patients after cardiovascular surgery). The ICU physicians input clinical information into the ICU database, which automatically registers medical charts as structured data. The study protocol was approved by the Ethics Committee of Hitachi General Hospital (2017–95). The need for informed consent was waived based on the study’s retrospective design.

### Study participants

We included all adult ICU patients (aged ≥20 years). According to the previous literature, we excluded patients with diabetic ketoacidosis or hyperosmolar hyperglycemic syndrome [9]. We also excluded patients with early ICU discharge (<48 hours) because of no need for glycemic control or early death regardless of glycemic control. In addition, when a patient had multiple ICU admissions, we included only the first ICU admission and excluded all subsequent ICU admissions.

### Data collection

We extracted patients’ physical information (age, sex, and body mass index [BMI]), disease severity (acute physiology score and acute physiology and chronic health evaluation [APACHE Ⅱ] score), disease category, and comorbidity according to the Charlson Comorbidity Index. APACHE II scores were calculated based on the worst values of vital signs and blood tests (as well as age and comorbidities) occurring during the first 24 hours of ICU admission. Using the measured blood glucose values, we calculated the mean blood glucose, glycemic variability, time above range over 220 mg/dL, time below range under 60 or 40 mg/dL, and TIR 70–180 mg/dL. We also calculated the coefficient of variation as the glycemic variability, which is defined as the standard deviation of blood glucose divided by the mean blood glucose. In addition, we extracted 28-day mortality, the length of ICU and hospital stay, and patient disposition. All of these data were extracted from electronic health records by using information system produced by TXP Medical Co., Ltd., which is linked to an electronic medical record system [14]. In addition, we have also reviewed medical records manually to confirm the extracted information.

### Glycemic control in the ICU

In the ICU of Hitachi General Hospital, staff members routinely collect blood gas every eight hours (three times per day) from patients with arterial line insertions. All blood glucose was measured using a blood gas analyzer (ABL90 FLEX, Radiometer, CPH, DK). Insulin was usually administered as a continuous infusion at a 1 U/mL concentration using a syringe pump with a target range of 70–180 mg/dL. The standard protocol of glycemic control in the ICU is shown in **S1 Fig**. Briefly, 1) we start continuous intravenous (IV) insulin when blood glucose is >180 mg/dL (for patients receiving continuous nutrition); 2) discontinue IV insulin if blood glucose falls <100 mg/dL; and 3) IV dextrose if blood glucose falls ≤70 mg/dL.

### Exposure and outcome

The primary exposure was TIR 70–180 mg/dL. The TIR was defined as the amount of time that blood glucose was between 70 and 180 mg/dL divided by the total ICU stay. The primary outcome was 28-day mortality.

### Statistical analysis

Summary statistics were used to describe the characteristics of the study participants as appropriate. Missing height and weight data occurred in 23.2% of these patients, but there were no missing data or marked outliers in other variables including blood glucose data.

According to the previous literature, analyses were stratified by HbA1c ≥6.5% (not the diagnosed diabetes) [9]. We examined the association between TIR ≥80% and 28-day mortality using two multivariable models: 1) logistic regression and 2) Cox proportional hazards models. In addition, we also examined the association between TIR and mortality using a logistic regression model in patients with HbA1c <6.5%, stratifying by the presence of diagnosed diabetes. We adjusted for age, sex, APACHE Ⅱ score, Charlson comorbidity index, and the primary diagnosis category (sepsis, cerebrovascular diseases, cardiac diseases, respiratory diseases, gastrointestinal diseases, trauma, postoperative, and others). Kaplan-Meier survival curves were graphed for these populations.

In the sensitivity analyses, first, we repeated the analyses using TIR as a 1) category with 10% incremental of TIR (<60%, 60%-69%, 70%-79%, 80%-89%, and ≥90%) and as a 2) category based on quartiles of patient distribution (<53%, 53%-80%, 81%-93%, and ≥94%; **S2 Fig**). Second, to assess the linear association of TIR with the outcome, we repeated the analyses using TIR (10% decremental) as a continuous variable. Third, we repeated the primary analysis using TIR during the first three days from the ICU admission, assuming that survived patients were more likely to have stable TIRs. In addition, to test the hypothesis that survived patients were more likely to have stable TIRs, we depicted the association of ICU length-of-stay with the median TIR and examined the correlation using Spearman’s rank test.

Furthermore, to clarify the association between the quality of glycemic control achieved as a result and mortality, we assessed the association between TIR 70–140 mg/dL and mortality using a logistic regression model in patients with HbA1c <6.5% managed with a target glycemic range of 70–180 mg/dL.

A two-sided p-value of <0.05 was considered statistically significant. Statistical analyses were conducted using R version 4.0.2. (The R Foundation for Statistical Computing).

## Results

### Patient characteristics

From 1 January 2016 through 31 October 2019, 2,288 patients were admitted to the ICU. Of these, we excluded 48 patients under the age of 20 years, 43 patients with diabetic ketoacidosis and hyperosmolar hyperglycemic syndrome, 893 patients those who were discharged from the ICU within 48 hours (808 were good recovery and 85 died), and 74 multiple ICU admissions. The remaining 1,230 patients were eligible for the analysis (**S3 Fig**). Their median age was 76 years; 65% were male, with median BMI of 22.1 (**Table 1**). The median HbA1c on admission was 5.9%; 250 patients (20.2%) had HbA1c ≥6.5%. No significant differences were found in age, BMI, comorbidity, or severity (e.g., APACHE Ⅱ score) between patients with HbA1c <6.5% and those with HbA1c ≥6.5%. Patients with HbA1c ≥6.5% had significantly higher mean blood glucose levels and glycemic variability than those with HbA1c <6.5%. Median TIR was 85.7% for patients with HbA1c <6.5% and 41.8% for patients with HbA1c ≥6.5% (p <0.001). Patients with HbA1c ≥6.5% had higher 28-day mortality than those with <6.5% (32.0% vs. 22.9%; p = 0.004).

**Table 1:** Patient characteristics, glycemic control metrics, and outcomes, according to HbA1c levels.

			All patients	HbA1c <6.5%	HbA1c ≥6.5%	P value
			(n = 1,230)	(n = 980)	(n = 250)	
Patient characteristics						
	Age, years		76 [64, 82]	75 [64, 82]	76 [64, 82]	0.49
	Male sex		797 (64.8)	618 (63.1)	179 (71.6)	0.01
	Body mass index		22.1 [20, 23]	22.1 [20, 23]	22.1 [21, 24]	0.08
	Acute physiology score		13 [9, 18]	13 [9, 18]	13 [9, 19]	0.26
	APACHE Ⅱ score		18 [13, 23]	18 [14, 23]	18.5 [13, 24]	0.32
	Charlson comorbidity index score		3 [2, 5]	3.5 [2, 5]	3 [2, 5]	0.13
	HbA1c, %		5.9 [5.4, 6.4]	5.7 [5.3, 6.0]	7.2 [6.8, 8.5]	<0.001
	Diagnosed Diabetes		376 (30.6)	209 (21.3)	167 (66.8)	<0.001
	Diagnosis					0.01
		Sepsis	310 (25.2)	233 (23.8)	77 (30.8)	
		Cerebrovascular diseases	122 (9.9)	102 (10.4)	20 (8.0)	
		Cardiac diseases	96 (7.8)	71 (7.2)	25 (10)	
		Cardiac arrest	126 (10.2)	91 (9.3)	35 (14)	
		Respiratory diseases	173 (14.1)	147 (15)	26 (10.4)	
		Gastrointestinal diseases	58 (4.7)	47 (4.8)	11 (4.4)	
		Trauma	63 (5.1)	49 (5.0)	14 (5.6)	
		Postoperative	46 (3.7)	39 (4.0)	7 (2.8)	
		Others	236 (19.2)	201 (20.5)	35 (14)	
Glycemic control metrics						
	The number of BG tests per day		3 [3, 3]	3 [3, 3]	3 [3, 3]	0.89
	Mean blood glucose, mg/dL		155 [136, 181]	149 [134, 170]	199 [161, 225]	<0.001
	Coefficient of variation, %		31.2 [20, 50]	28.6 [19, 43]	52.7 [30, 79]	<0.001
	Time above 220 mg/dL, %		0 [0, 20]	0 [0, 11]	31.3 [4, 48]	<0.001
	Hypoglycemia <70 mg/dL		116 (9.4)	82 (8.4)	34 (13.6)	0.02
	Hypoglycemia <40 mg/dL		54 (4.4)	35 (3.6)	19 (7.6)	0.009
	Time in range 70–180 mg/dL, %		80.7 [53, 93]	85.7 [65, 95]	41.8 [27, 76]	<0.001
	Time in range 70–180 mg/dL ≥80%		644 (52.4)	586 (59.8)	58 (23.2)	<0.001
Outcomes						
	ICU length-of-stay, days		6 [4, 10]	6 [4, 10]	6 [4, 10]	0.48
	Hospital length-of-stay, days		20 [11, 41]	20.5 [11, 41]	18.5 [10, 38]	0.49
	28-day mortality		304 (24.7)	224 (22.9)	80 (32.0)	0.004

Data are presented as median (interquartile range) or number (percentage). The P values are not adjusted for multiple comparisons.

APACHE, Acute Physiology and Chronic Health Evaluation; BG, Blood glucose; HbA1c, glycosylated hemoglobin; ICU, Intensive care unit.

### Association of TIR 70–180 mg/dL ≥80% with 28-day mortality

**Fig 1:**
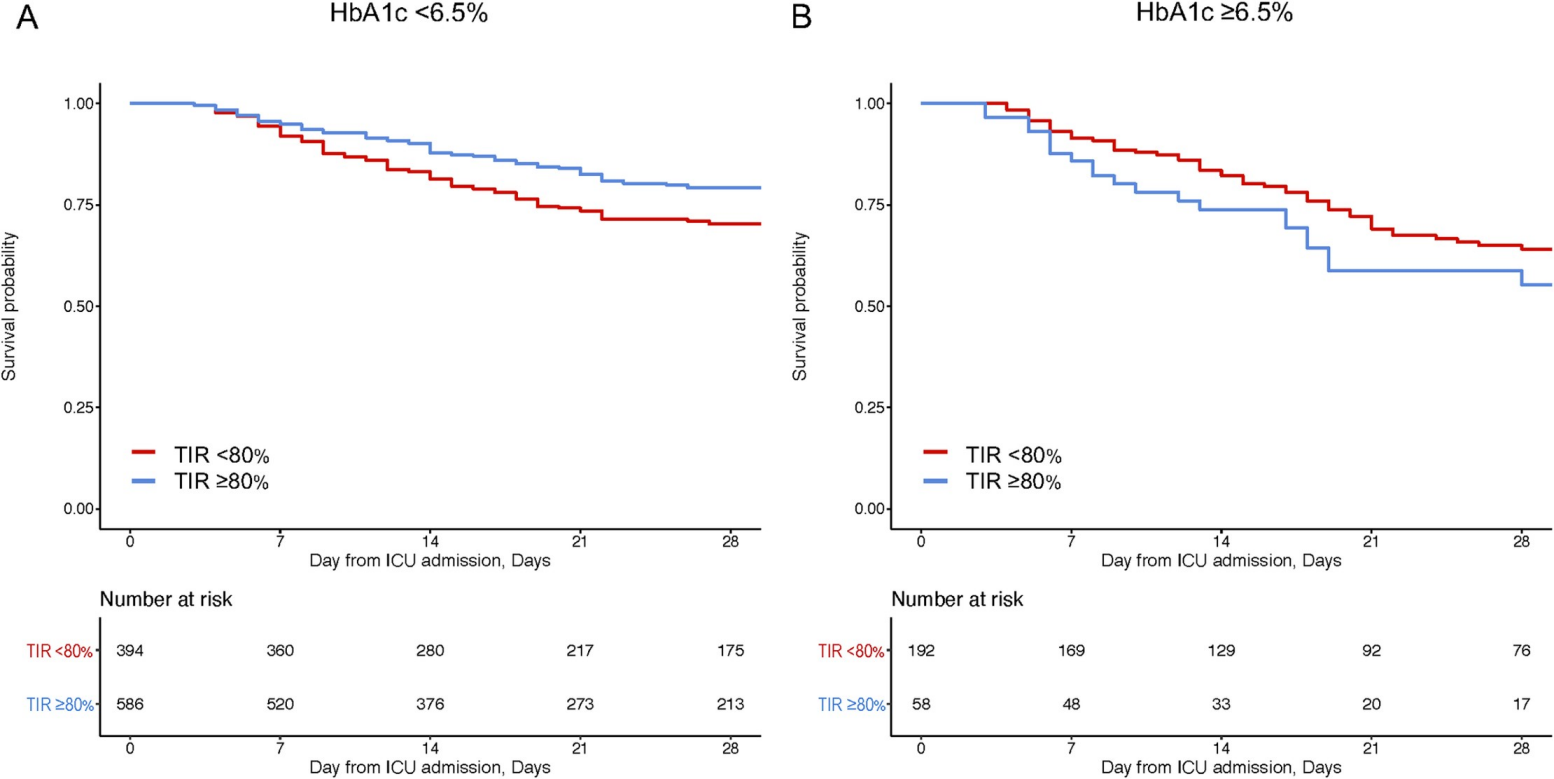
Association of time in range 70–180 mg/dL with 28-day survival according to HbA1c. Kaplan-Meier curves for patients with and without TIR <80%; TIR <80% (red) and TIR ≥80% (blue), in patients with HbA1c <6.5% (A), patients with HbA1c ≥6.5% (B). TIR, Time in range; HbA1c, glycosylated hemoglobin.

Compared with TIR ≥80%, TIR <80% was associated with the worse 28-day mortality in patients with HbA1c <6.5% (unadjusted odds ratio [OR], 2.23 [95%CI 1.65–3.01]; **Table 2** and **Fig 1**). This association remained significant in the multivariate logistic regression model with a similar effect size (adjusted OR, 2.21 [95%CI 1.60–3.05]). By contrast, in patients with HbA1c ≥6.5%, there was no significant association between TIR ≥80% and 28-day mortality in either unadjusted or adjusted logistic regression models. These associations of TIR with 28-day mortality were consistent with results obtained using Cox proportional hazards models (**S1 Table**). In patients with HbA1c <6.5%, TIR <80% was significantly associated with worse 28-day mortality than TIR ≥80%, regardless of the presence of diagnosed diabetes (**S4 Fig**). This association remained significant in the multivariable models (**S2 Table**).

**Table 2:** Associations between time in range 70–180 mg/dL and 28-day mortality using logistic regression models.

		HbA1c <6.5%				HbA1c ≥6.5%			
Time in range		Mortality / n	Mortality rate	Unadjusted OR	Adjusted OR*	Mortality / n	Mortality rate	Unadjusted OR	Adjusted OR*
				(95%CI)	(95%CI)			(95%CI)	(95%CI)
Time in range (threshold at 80%)									
	<80%	124 / 394	32%	2.23 (1.65–3.01)	2.21 (1.60–3.05)	59 / 192	31%	0.78 (0.42–1.45)	0.64 (0.30–1.37)
	≥80%	100 / 586	17%	1 (reference)	1 (reference)	21 / 58	36%	1 (reference)	1 (reference)
Time in range (10% incremental category)									
	<60%	78 / 205	38%	3.76 (2.53–5.58)	3.62 (2.36–5.53)	56 / 160	35%	1.77 (0.71–4.38)	1.19 (0.40–3.53)
	60%-69%	24 / 86	28%	2.40 (1.39–4.16)	2.39 (1.33–4.28)	1 / 13	8%	0.27 (0.03–2.49)	0.13 (0.01–1.42)
	70%-79%	22 / 103	21%	1.67 (0.96–2.88)	1.63 (0.92–2.91)	2 / 19	11%	0.39 (0.07–2.10)	0.14 (0.02–0.97)
	80%-89%	43 / 178	24%	1.96 (1.26–3.06)	1.83 (1.14–2.93)	14 / 28	50%	3.29 (1.07–10.1)	1.81 (0.46–7.06)
	≥90%	57 / 408	14%	1 (reference)	1 (reference)	7 / 30	23%	1 (reference)	1 (reference)
Time in range (10% decremental)				1.21 (1.14–1.29)	1.20 (1.12–1.28)			1.02 (0.94–1.12)	1.08 (0.96–1.21)
Time in range (quartile category)									
	Q1 (<53%)	60 / 156	39%	4.07 (2.55–6.48)	3.73 (2.26–6.14)	53 / 151	35%	1.71 (0.64–4.55)	1.21 (0.36–4.05)
	Q2 (53%-80%)	70 / 260	27%	2.42 (1.57–3.74)	2.28 (1.44–3.61)	6 / 44	14%	0.50 (0.14–1.76)	0.17 (0.04–0.76)
	Q3 (81%-93%)	55 / 268	21%	1.71 (1.09–2.67)	1.57 (0.98–2.53)	15 / 30	50%	3.17 (0.99–10.1)	2.06 (0.49–8.75)
	Q4 (≥94%)	39 / 296	13%	1 (reference)	1 (reference)	6 / 25	24%	1 (reference)	1 (reference)

*Logistic regression adjusted for age, sex, Charlson comorbidity index, APACHE Ⅱ score, and primary diagnosis category (sepsis, cerebrovascular diseases, cardiac diseases, cardiac arrest, respiratory diseases, gastrointestinal diseases, trauma, postoperative, and others).

APACHE, Acute Physiology and Chronic Health Evaluation; HbA1c, glycosylated hemoglobin; OR, odds ratio; CI, confidence interval.

### Association of TIR 70–180 mg/dL category with 28-day mortality

#### 10% incremental category

When using 10% incremental TIR as a categorical variable (<60%, 60%-69%, 70%-79%, 80%-89%, and ≥90%), the lower TIR was associated with the worse 28-day mortality compared to TIR ≥90% in patients with HbA1c <6.5% in both unadjusted and adjusted models (e.g., adjusted OR of TIR <60%, 3.62 [95%CI 2.36–5.53]; **Table 2**). In addition, there was a linear association between 10% TIR decrement and the increased risk of 28-day mortality (adjusted OR per 10% decrement, 1.20 [95%CI 1.12–1.28]). By contrast, no clear association was found between TIR and mortality in patients with HbA1c ≥6.5% in either unadjusted or adjusted logistic regression models. These relations of TIR with 28-day mortality were consistent with results obtained using Cox proportional hazards models (**S1 Table** and **S5 Fig**).

#### Quartile category

**Fig 2:**
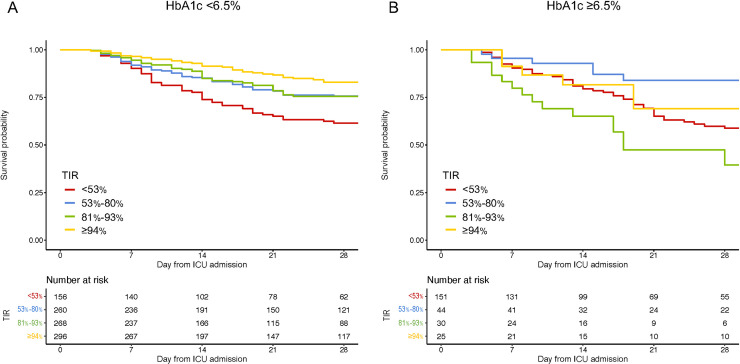
Association of time in range 70–180 mg/dL as a categorical variable (quartile) with 28-day survival according to HbA1c. Kaplan-Meier curves for patients with each quartile category of TIR; <53% (red), 53%-80% (blue), 81%-93% (green), and ≥94% (yellow), in patients with HbA1c <6.5% (A), patients with HbA1c ≥6.5% (B). TIR, Time in range; HbA1c, glycosylated hemoglobin.

When using TIR as a categorical variable based on a quartile of patients (<53%, 53%-80%, 81%-93%, and ≥94%), the association between the TIR category and 28-day mortality was similar to those using 10% incremental TIR as a categorical variable. For example, TIR <53% was associated with worse 28-day mortality compared to TIR ≥94% (adjusted OR 3.73 [95%CI 2.26–6.14]; **Table 2** and **Fig 2**).

### Sensitivity analyses using TIR 70–180 mg/dL of the first three days

**Fig 3:**
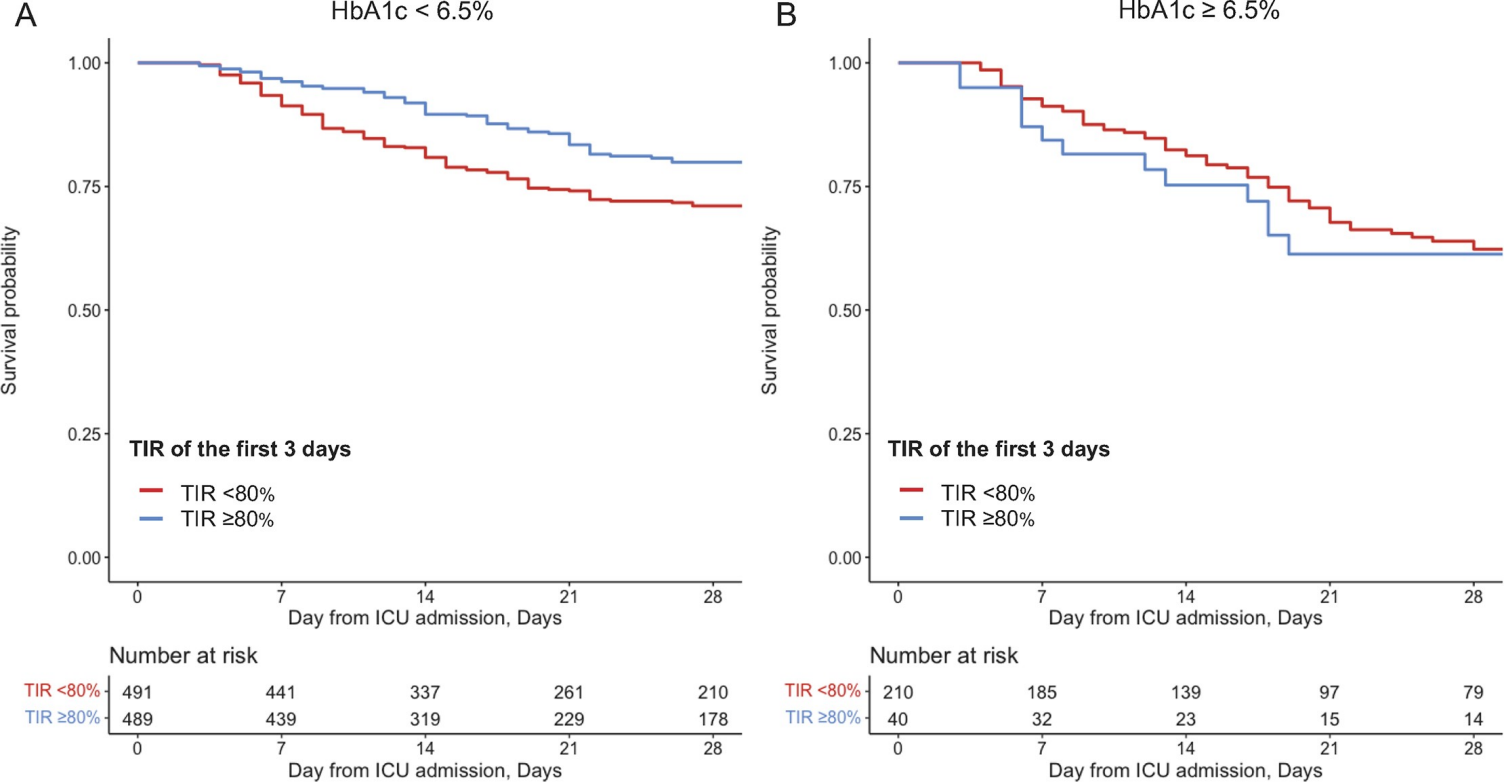
Association of time in range 70–180 mg/dL of the first three days with 28-day survival according to HbA1c. Kaplan-Meier curves for patients with and without TIR <80%; TIR <80% (red) and TIR ≥80% (blue), in patients with HbA1c <6.5% (A), patients with HbA1c ≥6.5% (B). TIR, Time in range; HbA1c, glycosylated hemoglobin.

In the analysis using TIR during the first three days from the ICU admission, the overall associations were consistent with those found from the primary analyses (**Fig 3**). For example, TIR <80% was associated with the worse 28-day mortality in patients with HbA1c <6.5% (e.g., adjusted OR of TIR <80%, 1.88 [95%CI 1.36–2.61]: **Table 3**). Similarly, the lower TIR was associated with the worse 28-day mortality in patients with HbA1c <6.5% (e.g., adjusted OR of TIR <60%, 2.02 [95%CI 1.33–3.06]; **Table 3**). In addition, as shown in the **S6 Fig**, patients with longer ICU length-of-stay were likely to have higher TIR (Spearman’s rank correlation coefficient of 0.55, p = 0.0019).

**Table 3:** Results of sensitivity analyses using time in range during the first three days.

		HbA1c <6.5%				HbA1c ≥6.5%			
Time in range		Mortality / n	Mortality rate	Unadjusted OR	Adjusted OR*	Mortality / n	Mortality rate	Unadjusted OR	Adjusted OR*
Time in range (threshold at 80%)									
	<80%	144 / 491	29%	2.12 (1.56–2.89)	1.88 (1.36–2.61)	67 / 210	32%	0.97 (0.47–2.00)	0.56 (0.23–1.37)
	≥80%	80 / 489	16%	1 (reference)	1 (reference)	13 / 40	33%	1 (reference)	1 (reference)
Time in range (10% incremental category)									
	<60%	84 / 261	32%	2.32 (1.57–3.42)	2.02 (1.33–3.06)	56 / 171	33%	1.32 (0.52–3.33)	0.65 (0.21–1.97)
	60%-69%	20 / 92	22%	1.36 (0.77–2.41)	1.16 (0.63–2.14)	4 / 15	27%	0.99 (0.23–4.15)	0.25 (0.05–1.37)
	70%-79%	40 / 138	29%	2.00 (1.25–3.19)	1.90 (1.15–3.13)	7 / 24	29%	1.12 (0.32–3.84)	0.37 (0.08–1.68)
	80%-89%	25 / 165	15%	0.87 (0.52–1.46)	0.88 (0.51–1.51)	6 / 14	43%	2.04 (0.52–8.00)	1.09 (0.20–6.07)
	≥90%	55 / 324	17%	1 (reference)	1 (reference)	7 / 26	27%	1 (reference)	1 (reference)
Time in range (10% decremental)				1.11 (1.05–1.16)	1.09 (1.03–1.15)			1.01 (0.93–1.10)	1.01 (0.91–1.12)
Time in range (quartile category)									
	Q1 (<53%)	61 / 187	33%	2.28 (1.42–3.65)	2.07 (1.25–3.43)	50 / 153	33%	1.34 (0.54–3.66)	0.66 (0.21–2.05)
	Q2 (53%-80%)	83 / 304	27%	1.91 (1.27–2.89)	1.61 (1.04–2.50)	17 / 57	30%	1.19 (0.44–3.50)	0.52 (0.15–1.76)
	Q3 (81%-93%)	25 / 165	15%	1.34 (0.90–1.99)	1.52 (0.86–2.01)	6 / 14	43%	1.41 (0.48–4.39)	0.56 (0.15–2.10)
	Q4 (≥94%)	55 / 324	17%	1 (reference)	1 (reference)	7 / 26	27%	1 (reference)	1 (reference)

*Logistic regression adjusted for age, sex, Charlson comorbidity index, APACHE Ⅱ score, and primary diagnosis category (sepsis, cerebrovascular diseases, cardiac diseases, cardiac arrest, respiratory diseases, gastrointestinal diseases, trauma, postoperative, and others).

APACHE, Acute Physiology and Chronic Health Evaluation; HbA1c, glycosylated hemoglobin; OR, odds ratio; CI, confidence interval.

### Association of TIR 70–140 mg/dL with 28-day mortality in patients with HbA1c <6.5%

Compared with TIR 70–140 mg/dL ≥80%, TIR 70–140 mg/dL <80% was associated with the worse 28-day mortality in patients with HbA1c <6.5% (unadjusted OR, 3.54 [95%CI 2.10–6.39]; **S3 Table**). This association remained significant in the multivariate logistic regression model with a similar effect size (adjusted OR, 3.02 [95%CI 1.75–5.56]; **S3 Table**). When using 10% incremental TIR 70–140 mg/dL as a categorical variable (<60%, 60%-69%, 70%-79%, 80%-89%, and ≥90%), the lower TIR was associated with the worse 28-day mortality compared to TIR ≥90% in both unadjusted and adjusted models (e.g., adjusted OR of TIR <60%, 2.72 [95%CI 1.18–7.40]; **S3 Table**). Also, when using TIR as a categorical variable based on a quartile of patients (<20%, 20%-43%, 44%-64%, and ≥65%), the association between the TIR category and 28-day mortality was similar to those using 10% incremental TIR as a categorical variable.

## Discussion

This study of 1,230 ICU patients found that TIR 70–180 mg/dL was associated with 28-day mortality in critically ill patients with HbA1c <6.5%, although no apparent association was found in those with HbA1c ≥6.5%. While the causal relationship cannot be determined, based on the analyses conducted using several cut-off values, the optimal range of TIR may be >90%, or >80% is also reasonable for patients with HbA1c <6.5%. Furthermore, similar findings obtained using TIR during the first three days support the use of TIR as a prognostic marker for critically ill patients.

The observed relation of higher TIR with a lower mortality in critically ill patients was consistent with those of earlier studies [7, 9–11]. Krinsley et al. examined mortality between the high-TIR and low-TIR groups and found significantly lower mortality in the high-TIR group than in the low-TIR group among non-diabetic patients (8.5% vs. 15.8%, p<0.001), although no difference was found in mortality between groups among diabetic patients (16.1% vs. 14.4%, p = 0.60) [7]. Similarly, Lanspa et al. found that the relation between TIR >80% and mortality was likely to depend on HbA1c rather than on whether the patient had diabetes mellitus [9]. In fact, in this study, we also analyzed patients with HbA1c<6.5% stratified by the presence of diagnosed diabetes, and found that TIR>80% was significantly associated with mortality, regardless of the diagnosis of diabetes (**S2 Table** and **S4 Fig**).

Earlier studies of critically ill patients have described an association between TIR and mortality with a target range of 70–140 mg/dL for blood glucose [7, 9]. Whereas these earlier studies provided concrete evidence on the glucose management for ICU patients, the current national guidelines recommend the more liberal upper target glucose level of 180 mg/dL [13]. We found that TIR 70–140 mg/dL <80% was significantly associated with mortality, and in patients with HbA1c <6.5%, 28-day mortality in patients with TIR 70–140 mg/dL ≥80% was lower than those in patients with TIR 70–180 mg/dL ≥80% (9% vs. 17%, **Table 2** and **S3 Table**). The direct comparison may be difficult because the target range of glycemic control was 70–180 mg/dL in our hospital (not 70–140 mg/dL). However, when assessing the association between glycemic control and mortality, from a physiological standpoint, the resulting level of glycemia achieved may be more important than how we set the glycemic target range [10].

Moreover, few studies have addressed survival bias, by which surviving patients were more likely to have stable TIR [15]. Although we hypothesized that survived patients were more likely to have stable TIR, this survivorship bias might be affected by the glucose management protocol and the definition of TIR. Indeed, a previous study reported that diabetic patients (who are more likely to be out-of-range) were more likely to have longer time on insulin therapy compared to non-diabetic patients [16]. Nevertheless, the observed correlation between the ICU length-of-stay and the median TIR (**S6 Fig**) and the results using TIR during the first three days should support our inference.

Critically ill patients can easily become hyperglycemic because of various factors [17, 18]. Glucose is a pro-inflammatory mediator with mechanisms such as increasing plasma interleukin-8 levels [19], increasing nuclear factor kappa-light-chain-enhancer of activated B cells [20], and increasing plasma matrix metalloproteinase (MMP) -2 and MMP-9 levels [21]. Although hyperglycemia rarely causes death directly, prolonged hyperglycemia can contribute to morbid conditions such as sepsis and post-intensive care syndrome by causing a toxic cellular environment and impaired immune function [22]. By contrast, hypoglycemia is an iatrogenic complication that should be avoided. The NICE-SUGAR Study investigators showed that moderate hypoglycemia (40–70 mg/dL) and severe hypoglycemia (<40 mg/dL) were both independent risk factors of death (moderate: HR 1.41 [95%CI 1.21–1.62], severe: HR 2.10 [95%CI 1.59–2.77]) [23]. Hypoglycemia presents an independent risk of death, as well as risks of convulsions and arrhythmias [24, 25]. The management of glycemic control to avoid hyperglycemia and hypoglycemia, as clinicians have attempted for many decades, is nothing less than maintaining high TIR management. Therefore, the finding that high TIR was associated significantly with low mortality was clinically plausible.

Importantly, the association between TIR 70–180 mg/dL and mortality was found only in patients with HbA1c <6.5% on admission: not in those with ≥6.5%. Several studies have revealed similar associations [7, 9]. Indeed, unlike patients with diabetes mellitus, TIR 70–140 mg/dL >80% was not associated with mortality in critically ill patients without diabetes mellitus [7]. Possible explanations include that patients with HbA1c ≥6.5% on admission were more likely to have been chronically hyperglycemic. Chronic hyperglycemia may attenuate the extent of cellular toxicity in acute hyperglycemia by downregulating the glucose transporter (GLUT) 1 and GLUT 4 transporters [26]. In addition, patients with a high HbA1c on admission may be less likely to be harmed by a low TIR because they are relatively less harmed by exposure to hyperglycemia [27].

### Potential limitations

There are several potential limitations in this study. First, unmeasured factors related to assessment of the association of TIR with outcomes may exist because of the nature of retrospective design. Second, this study calculated TIR using glucose measured using a blood gas analyzer every eight hours. Compared with previous studies, our insulin protocol requires fewer blood glucose measurements per day (three times per day). Because glycemic variability and control measures depend on the frequency of measurements, the lower frequency might result in wider tolerances, looser control, and possibly more unmeasured glycemic variation [7, 9]. Third, there might be a time-varying confounding and exposure-covariate feedback. That is, the lower TIR does not solely depend on only glucose management but also patient condition at each time. Fourth, of the total 18 ICU beds in our hospital, 10 contain beds with a 4:1 patient-nurse ratio, which may affect the ICU’s ability to provide safe and effective blood glucose control. Fifth, because this study was conducted at a single tertiary care institution in Japan, and the patients included may not reflect the Japanese population. Thus, the generalizability of the findings may be limited. Yet, these findings were consistent with previous studies [7, 13] and the observed association may not be substantially varied across hospitals.

## Conclusions

We found that lower TIR 70–180 mg/dL was associated with a higher 28-day mortality in critically ill patients with HbA1c <6.5 mg/dl, whereas there was no consistent association in patients with HbA1c ≥6.5%. Further validation studies are needed, but for glycemic control at a range of 70–180 mg/dL, TIR ≥90%, at least ≥80%, may be the target for critically ill patients with HbA1c <6.5%.

## Supporting information

S1 Fig The insulin protocol of the Hitachi General Hospital. BG, blood glucose; ICU, intensive care unit.
(TIFF)

S2 Fig The distribution of time in range in patients admitted to the intensive care unit. The quartiles of TIR 70–180 mg/dL were as follow, lower quartile, 53.2%; median, 80.7%; upper quartile, 93.3%. TIR, Time in range.
(TIFF)

S3 Fig Study flow. ICU, intensive care unit; DKA, diabetic ketoacidosis; HHS, hyperosmolar hyperglycemic syndrome.
(TIFF)

S4 Fig Association between time in range and 28-day mortality according to the HbA1c and diagnosis of diabetes. Kaplan-Meier curves for patients with and without TIR <80%; TIR <80% (red) and TIR ≥80% (blue), in patients with HbA1c <6.5% and non-DM (A), patients with HbA1c <6.5% and DM (B). HbA1c, glycosylated hemoglobin; DM, diabetes mellitus; TIR, time in range.
(TIFF)

S5 Fig Association of time in range as 10% incremental category with 28-day mortality according to HbA1c. Kaplan-Meier curves for patients with each 10% incremental category of TIR; <60% (red), 60%-69% (blue), 70%-79% (green), 80%-89% (yellow), and ≥90% (gray), in patients with HbA1c <6.5% (A), patients with HbA1c ≥6.5% (B). TIR, Time in range; HbA1c, glycosylated hemoglobin.
(TIFF)

S6 Fig Association of ICU length-of-stay with median time in range 70–180 mg/dL. There was a weak correlation between the ICU length-of-stay and median TIR 70–180 mg/dL (Spearman’s rank correlation coefficient of 0.55, p = 0.0019). TIR, time in range; ICU, intensive care unit.
(TIFF)

S1 Table Associations between time in range 70–180 mg/dL and 28-day mortality using Cox proportional hazards model. *Cox proportional hazards model adjusted for age, sex, Charlson comorbidity index, APACHE Ⅱ score, and primary diagnosis category (sepsis, cerebrovascular diseases, cardiac diseases, cardiac arrest, respiratory diseases, gastrointestinal diseases, trauma, postoperative, and others). APACHE, Acute Physiology and Chronic Health Evaluation; HbA1c, glycosylated hemoglobin; HR, hazard ratio.
(DOCX)

S2 Table Association between time in range 70–180 mg/dL and 28-day mortality in patients with HbA1c <6.5% according to the presence of the diagnosed diabetes. *Logistic regression adjusted for age, sex, Charlson comorbidity index, APACHE Ⅱ score, and primary diagnosis category (sepsis, cerebrovascular diseases, cardiac diseases, cardiac arrest, respiratory diseases, gastrointestinal diseases, trauma, postoperative, and others). APACHE, Acute Physiology and Chronic Health Evaluation; HbA1c, glycosylated hemoglobin; OR, odds ratio; CI, confidence interval.
(DOCX)

S3 Table Association between time in range 70–140 mg/dL and 28-day mortality in patients with HbA1c <6.5%. *Logistic regression adjusted for age, sex, Charlson comorbidity index, APACHE Ⅱ score, and primary diagnosis category (sepsis, cerebrovascular diseases, cardiac diseases, cardiac arrest, respiratory diseases, gastrointestinal diseases, trauma, postoperative, and others). APACHE, Acute Physiology and Chronic Health Evaluation; HbA1c, glycosylated hemoglobin; CI, confidence interval.
(DOCX)
