# Supplementary figures and images for: Time in blood glucose range 70 to 180 mg/dL and survival rate in critically ill patients: A retrospective cohort study
Source: PLoS One. 2021 May 27;16(5):e0252158. doi: 10.1371/journal.pone.0252158 (PMC8158903; doi:10.1371/journal.pone.0252158)

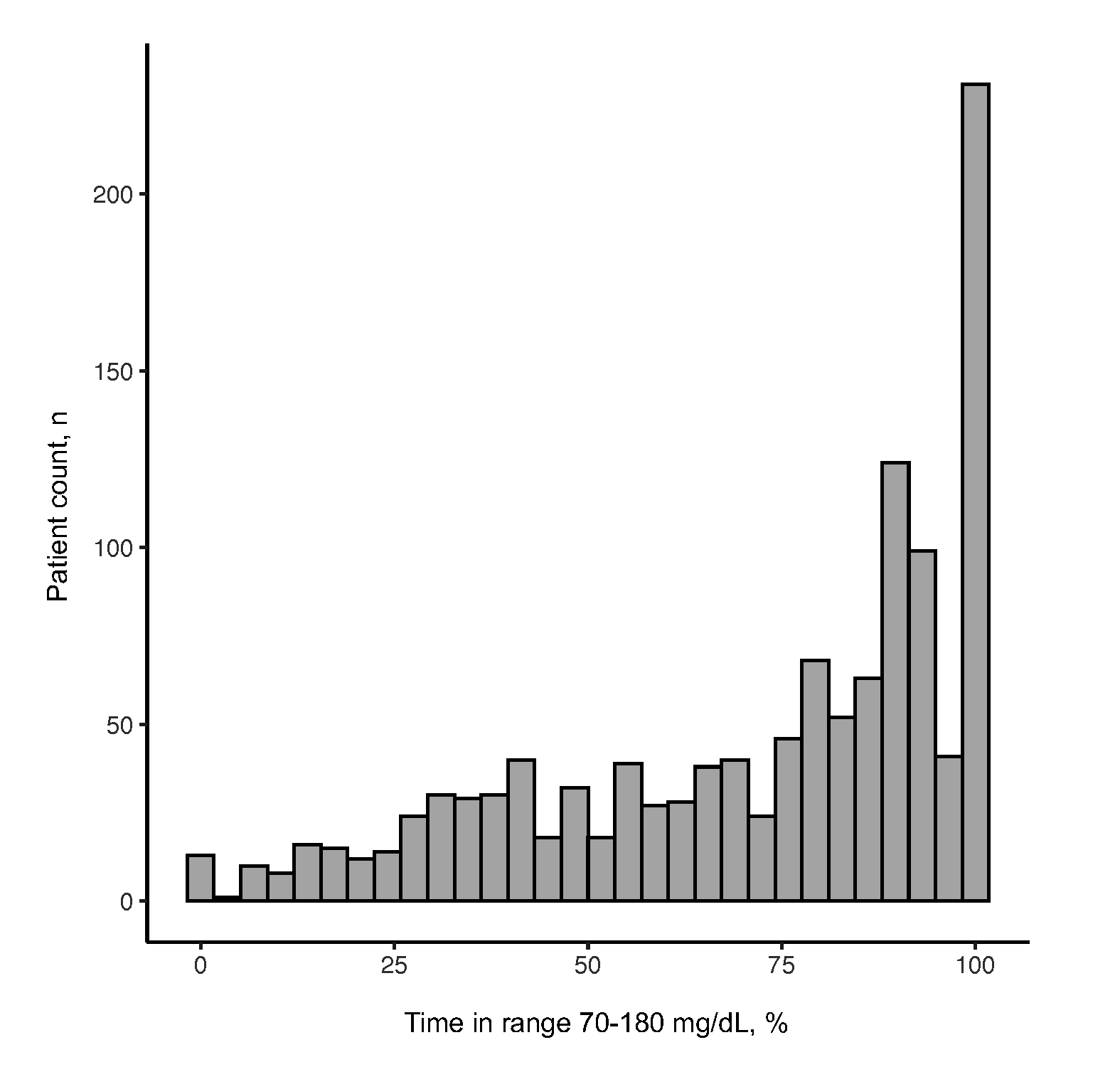

Supplement: S2 Fig — The quartiles of TIR 70–180 mg/dL were as follow, lower quartile, 53.2%; median, 80.7%; upper quartile, 93.3%. TIR, Time in range. (TIFF) [file pone.0252158.s002.tiff]

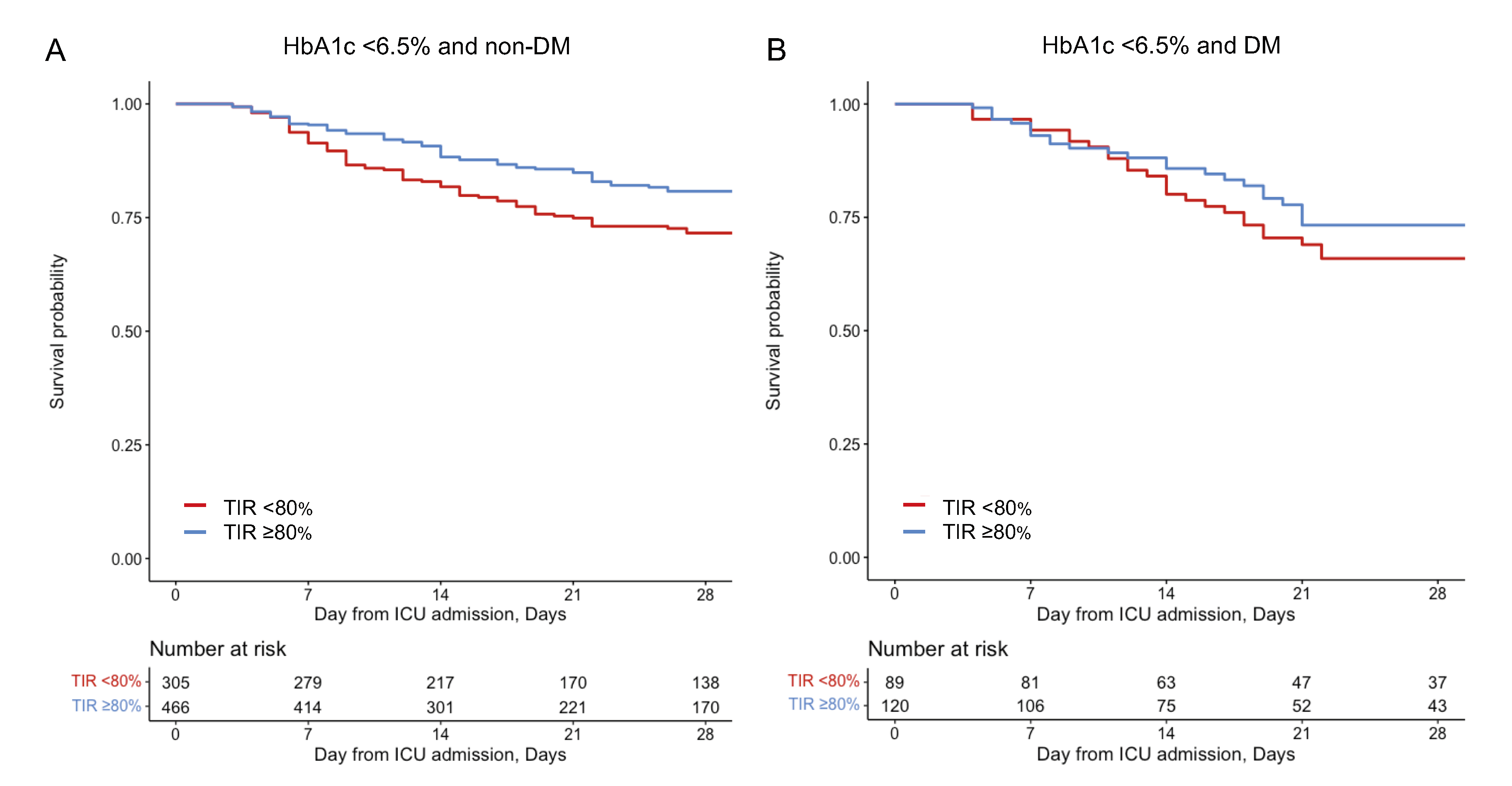

Supplement: S4 Fig — Kaplan-Meier curves for patients with and without TIR <80%; TIR <80% (red) and TIR ≥80% (blue), in patients with HbA1c <6.5% and non-DM (A), patients with HbA1c <6.5% and DM (B). HbA1c, glycosylated hemoglobin; DM, diabetes mellitus; TIR, time in range. (TIFF) [file pone.0252158.s004.tiff]

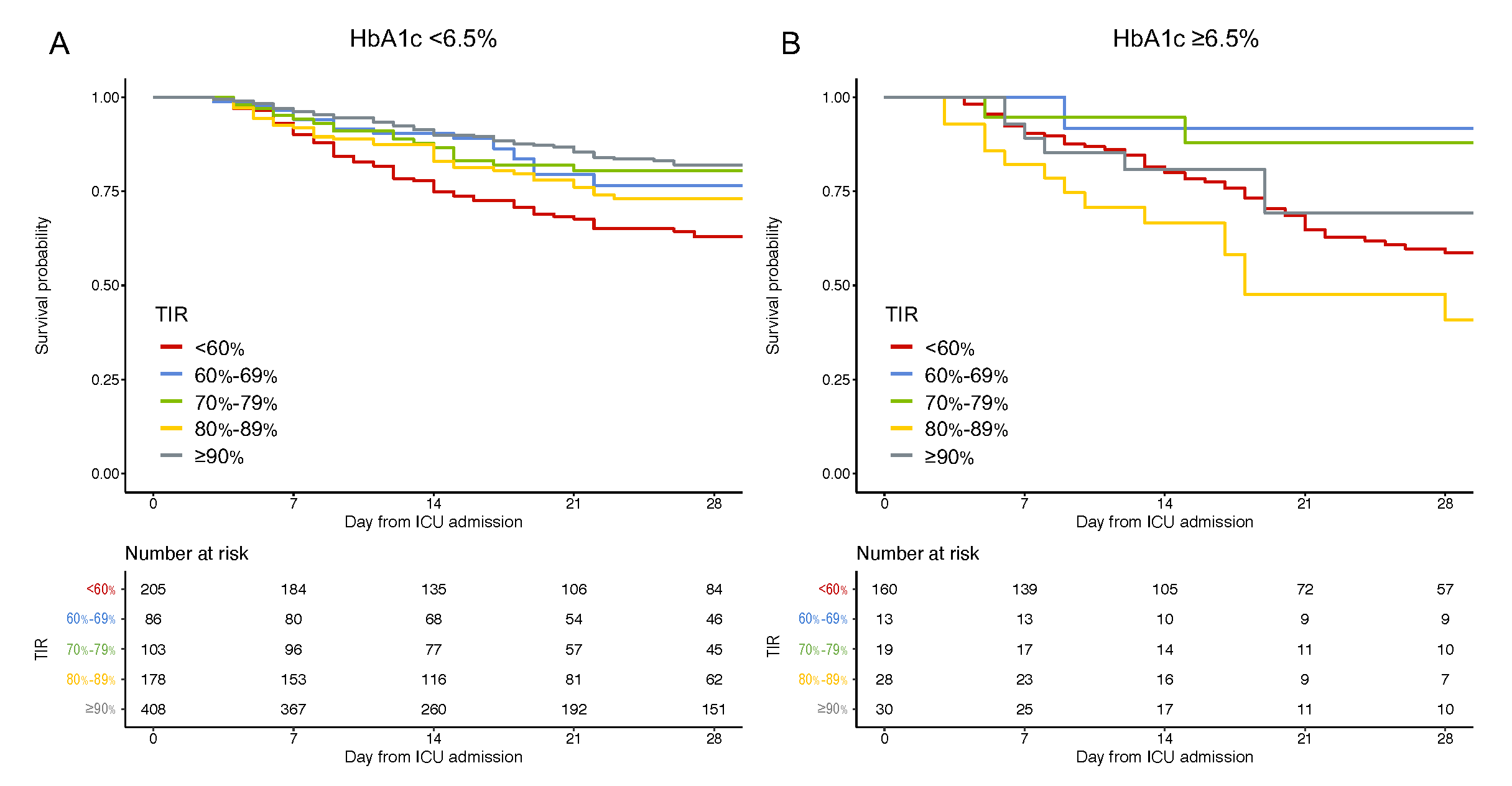

Supplement: S5 Fig — Kaplan-Meier curves for patients with each 10% incremental category of TIR; <60% (red), 60%-69% (blue), 70%-79% (green), 80%-89% (yellow), and ≥90% (gray), in patients with HbA1c <6.5% (A), patients with HbA1c ≥6.5% (B). TIR, Time in range; HbA1c, glycosylated hemoglobin. (TIFF) [file pone.0252158.s005.tiff]

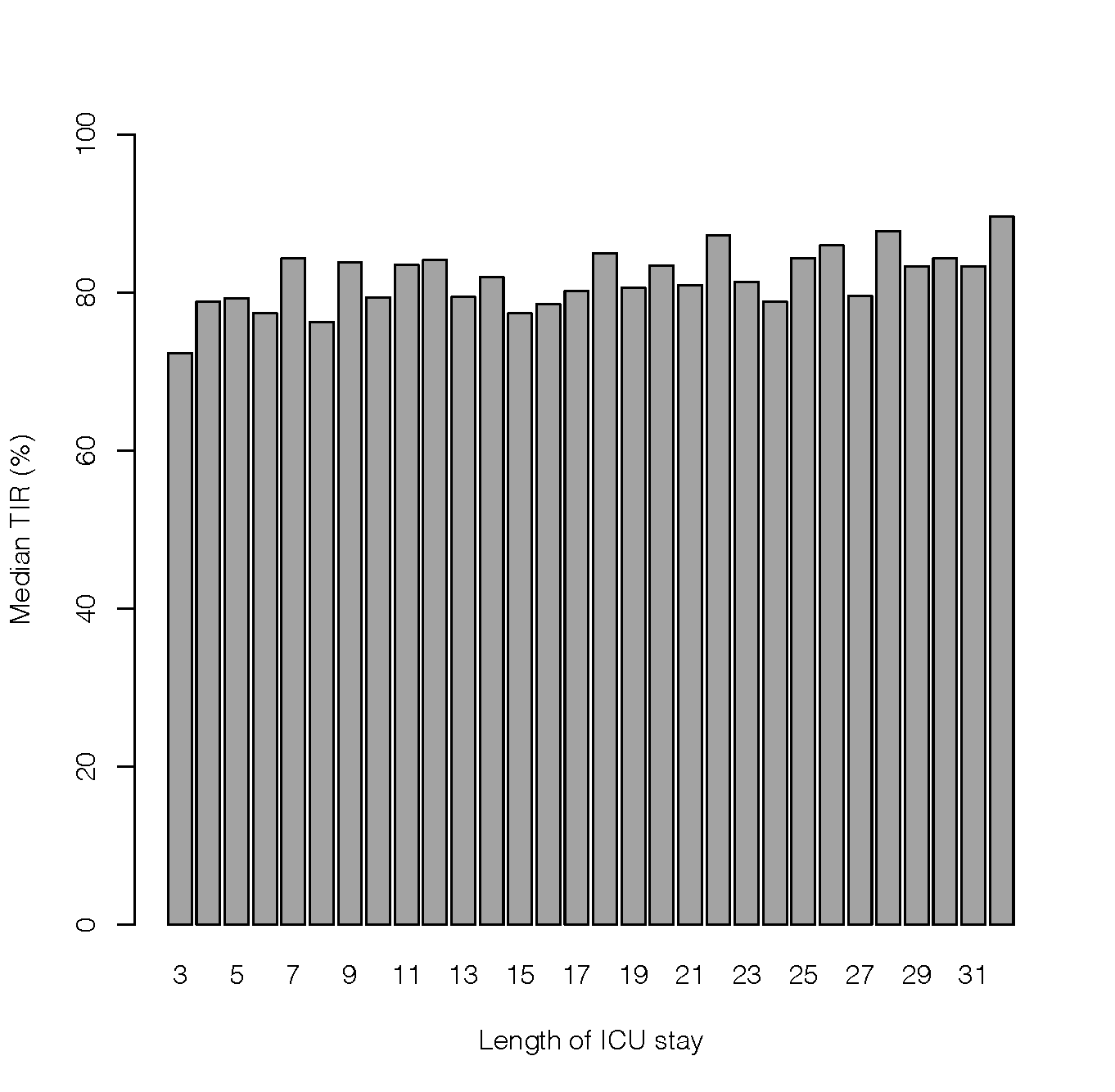

Supplement: S6 Fig — There was a weak correlation between the ICU length-of-stay and median TIR 70–180 mg/dL (Spearman’s rank correlation coefficient of 0.55, p = 0.0019). TIR, time in range; ICU, intensive care unit. (TIFF) [file pone.0252158.s006.tiff]
