# Supplementary material for: Time in blood glucose range 70 to 180 mg/dL and survival rate in critically ill patients: A retrospective cohort study
Source: PLoS One. 2021 May 27;16(5):e0252158. doi: 10.1371/journal.pone.0252158 (PMC8158903; doi:10.1371/journal.pone.0252158)
Supplement: S1 Table — *Cox proportional hazards model adjusted for age, sex, Charlson comorbidity index, APACHE Ⅱ score, and primary diagnosis category (sepsis, cerebrovascular diseases, cardiac diseases, cardiac arrest, respiratory diseases, gastrointestinal diseases, trauma, postoperative, and others). APACHE, Acute Physiology and Chronic Health Evaluation; HbA1c, glycosylated hemoglobin; HR, hazard ratio. (DOCX) [file pone.0252158.s007.docx]

**S1** **Table. Associations between time in range 70-180 mg/dL and 28-day mortality using Cox proportional hazards model**

|  |  | HbA1c <6.5% | | | |  | HbA1c ≥6.5% | | | |
| --- | --- | --- | --- | --- | --- | --- | --- | --- | --- | --- |
| Time in range | | Mortality / n | Mortality rate | Unadjusted HR | Adjusted HR† |  | Mortality / n | Mortality rate | Unadjusted HR | Adjusted HR† |
| Time in range (threshold at 80%) | |  |  |  |  |  |  |  |  |  |
|  | <80% | 124 / 394 | 32% | 1.68 (1.29-2.18)*** | 1.67 (1.27-2.18)*** |  | 59 / 192 | 31% | 0.74 (0.45-1.21) | 0.66 (0.39-1.11) |
|  | ≥80% | 100 / 586 | 17% | 1 (reference) | 1 (reference) |  | 21 / 58 | 36% | 1 (reference) | 1 (reference) |
| Time in range (10% incremental category) | |  |  |  |  |  |  |  |  |  |
|  | <60% | 78 / 205 | 38% | 2.62 (1.86-3.69)*** | 2.45 (1.73-3.46)*** |  | 56 / 160 | 35% | 1.41 (0.64-3.090 | 1.19 (0.52-2.72) |
|  | 60%-69% | 24 / 86 | 28% | 1.68 (1.04-2.71)* | 1.55 (0.96-2.51) |  | 1 / 13 | 8% | 0.23 (0.03-1.90) | 0.19 (0.02-1.54) |
|  | 70%-79% | 22 / 103 | 21% | 1.38 (0.84-2.26) | 1.35 (0.82-2.21) |  | 2 / 19 | 11% | 0.37 (0.08-1.78) | 0.26 (0.05-1.27) |
|  | 80%-89% | 43 / 178 | 24% | 1.79 (1.21-2.66)** | 1.50 (1.00-2.24)* |  | 14 / 28 | 50% | 2.36 (0.95-5.85) | 1.89 (0.74-4.82) |
|  | ≥90% | 57 / 408 | 14% | 1 (reference) | 1 (reference) |  | 7 / 30 | 23% | 1 (reference) | 1 (reference) |
| Time in range (10% decremental) | |  |  | 1.15 (1.09-1.21)*** | 1.14 (1.08-1.20)*** |  |  |  | 1.02 (0.95-1.11) | 1.06 (0.97-1.16) |
| Time in range (quartile category) | |  |  |  |  |  |  |  |  |  |
|  | Q1 (<53%) | 60 / 156 | 39% | 2.96 (1.98-4.42)*** | 2.78 (1.85-4.18)*** |  | 53 / 151 | 35% | 1.45 (0.62-3.37) | 1.23 (0.51-2.96) |
|  | Q2 (53%-80%) | 70 / 260 | 27% | 1.90 (1.29-2.81)*** | 1.70 (1.14-2.52)** |  | 6 / 44 | 14% | 0.50 (0.16-1.54) | 0.35 (0.11-1.11) |
|  | Q3 (81%-93%) | 55 / 268 | 21% | 1.71 (1.14-2.58)** | 1.52 (1.00-2.30)* |  | 15 / 30 | 50% | 2.55 (0.99-6.59)* | 1.98 (0.75-5.25) |
|  | Q4 (≥94%) | 39 / 296 | 13% | 1 (reference) | 1 (reference) |  | 6 / 25 | 24% | 1 (reference) | 1 (reference) |

* P value <0.05; ** P value <0.01; *** P value <0.001.

†Cox proportional hazards model adjusted for age, sex, Charlson comorbidity index, APACHE Ⅱ score, and primary diagnosis category (sepsis, cerebrovascular diseases, cardiac diseases, cardiac arrest, respiratory diseases, gastrointestinal diseases, trauma, postoperative, and others). APACHE, Acute Physiology and Chronic Health Evaluation; HbA1c, glycosylated hemoglobin; HR, hazard ratio.
