# Supplementary material for: Time in blood glucose range 70 to 180 mg/dL and survival rate in critically ill patients: A retrospective cohort study
Source: PLoS One. 2021 May 27;16(5):e0252158. doi: 10.1371/journal.pone.0252158 (PMC8158903; doi:10.1371/journal.pone.0252158)
Supplement: S2 Table — *Logistic regression adjusted for age, sex, Charlson comorbidity index, APACHE Ⅱ score, and primary diagnosis category (sepsis, cerebrovascular diseases, cardiac diseases, cardiac arrest, respiratory diseases, gastrointestinal diseases, trauma, postoperative, and others). APACHE, Acute Physiology and Chronic Health Evaluation; HbA1c, glycosylated hemoglobin; OR, odds ratio; CI, confidence interval. (DOCX) [file pone.0252158.s008.docx]

**S2 Table. Association between time in range 70-180 mg/dL and 28-day mortality in patients with HbA1c <6.5% according to the presence of the diagnosed diabetes**

|  |  | HbA1c <6.5% and non-diabetes | | | |  | HbA1c <6.5% and diabetes | | | |
| --- | --- | --- | --- | --- | --- | --- | --- | --- | --- | --- |
| Time in range | | Mortality / n | Mortality rate | Unadjusted OR | Adjusted OR* |  | Mortality / n | Mortality rate | Unadjusted OR | Adjusted OR* |
| Time in range (threshold at 80%) | |  |  |  |  |  |  |  |  |  |
|  | <80% | 93 / 305 | 31% | 2.29 (1.62-3.24) | 2.13(1.47-3.11) |  | 31 / 89 | 35% | 2.02 (1.04-3.96) | 2.37 (1.21-4.77) |
|  | ≥80% | 75 / 466 | 16% | 1 (reference) | 1 (reference) |  | 25 / 120 | 21% | 1 (reference) | 1 (reference) |
| Time in range (10% incremental category) | |  |  |  |  |  |  |  |  |  |
|  | <60% | 58 / 160 | 36% | 3.69 (2.35-5.84) | 3.34 (2.04-5.51) |  | 20 / 45 | 44% | 4.11 (1.83-9.53) | 5.08 (2.08-13.0) |
|  | 60%-69% | 18 / 63 | 29% | 2.60 (1.35-4.84) | 2.65 (1.32-5.18) |  | 6 / 23 | 26% | 1.81 (0.57-5.28) | 1.81 (0.53-5.81) |
|  | 70%-79% | 17 / 82 | 21% | 1.70 (0.89-3.12) | 1.51 (0.77-2.89) |  | 5 / 21 | 24% | 1.61 (0.47-4.91) | 1.71 (0.46-5.67) |
|  | 80%-89% | 32 / 144 | 22% | 1.85 (1.11-3.07) | 1.73 (1.00-2.98) |  | 11 / 34 | 32% | 2.45 (0.97-6.18) | 2.16 (0.78-5.96) |
|  | ≥90% | 43 / 322 | 13% | 1 (reference) | 1 (reference) |  | 14 / 86 | 16% | 1 (reference) | 1 (reference) |
| Time in range (quartile category) | |  |  |  |  |  |  |  |  |  |
|  | Q1 (<53%) | 46 / 125 | 37% | 4.20 (2.47-7.25) | 3.66 (1.85-4.18) |  | 14 / 31 | 37% | 4.11 (1.59-11.0) | 4.18 (1.51-12.0) |
|  | Q2 (53%-80%) | 51 / 196 | 26% | 2.54 (1.54-4.26) | 2.38 (1.40-4.14) |  | 19 / 64 | 30% | 2.11 (0.92-5.02) | 1.97 (0.82-4.97) |
|  | Q3 (81%-93%) | 43 / 220 | 20% | 1.75 (1.05-2.96) | 1.66 (0.96-2.91) |  | 12 / 48 | 25% | 1.67 (0.66-4.24) | 1.27 (0.47-3.46) |
|  | Q4 (≥94%) | 28 / 230 | 13% | 1 (reference) | 1 (reference) |  | 11 / 66 | 17% | 1 (reference) | 1 (reference) |

*Logistic regression adjusted for age, sex, Charlson comorbidity index, APACHE Ⅱ score, and primary diagnosis category (sepsis, cerebrovascular diseases, cardiac diseases, cardiac arrest, respiratory diseases, gastrointestinal diseases, trauma, postoperative, and others).

APACHE, Acute Physiology and Chronic Health Evaluation; HbA1c, glycosylated hemoglobin; OR, odds ratio.
