# Supplementary material for: Time in blood glucose range 70 to 180 mg/dL and survival rate in critically ill patients: A retrospective cohort study
Source: PLoS One. 2021 May 27;16(5):e0252158. doi: 10.1371/journal.pone.0252158 (PMC8158903; doi:10.1371/journal.pone.0252158)
Supplement: S3 Table — *Logistic regression adjusted for age, sex, Charlson comorbidity index, APACHE Ⅱ score, and primary diagnosis category (sepsis, cerebrovascular diseases, cardiac diseases, cardiac arrest, respiratory diseases, gastrointestinal diseases, trauma, postoperative, and others). APACHE, Acute Physiology and Chronic Health Evaluation; HbA1c, glycosylated hemoglobin; CI, confidence interval. (DOCX) [file pone.0252158.s009.docx]

**S3 Table. Association between time in range 70-140 mg/dL and 28-day mortality in patients with HbA1c <6.5%**

|  |  | Patients with HbA1c <6.5% | | | |
| --- | --- | --- | --- | --- | --- |
| Time in range 70-140 mg/dL | | Mortality / n | Mortality rate | Unadjusted odds ratio | Adjusted odds ratio* |
| Time in range (threshold at 80%) | |  |  |  |  |
|  | <80% | 206 / 812 | 26% | 3.54 (2.10-6.39) | 3.02 (1.75-5.56) |
|  | ≥80% | 15 / 168 | 9% | 1 (reference) | 1 (reference) |
| Time in range (10% incremental category) | |  |  |  |  |
|  | <60% | 164 / 586 | 28% | 3.30 (2.35-5.84) | 2.72 (1.18-7.40) |
|  | 60%-69% | 32 / 165 | 19% | 2.05 (1.35-4.84) | 1.81 (0.73-5.21) |
|  | 70%-79% | 13 / 61 | 21% | 2.30 (0.89-3.12) | 2.01 (0.68-6.44) |
|  | 80%-89% | 9 / 111 | 8% | 0.75 (1.11-3.07) | 0.73 (0.24-2.36) |
|  | ≥90% | 6 / 57 | 10% | 1 (reference) | 1 (reference) |
| Time in range (quartile category) | |  |  |  |  |
|  | Q1 (<20%) | 62 / 173 | 36% | 4.03 (2.54-6.52) | 3.59 (2.19-6.00) |
|  | Q2 (20%-43%) | 66 / 248 | 27% | 2.62 (1.68-4.15) | 2.28 (1.43-3.70) |
|  | Q3 (44%-64%) | 61 / 271 | 23% | 2.10 (1.34-3.33) | 2.04 (1.27-3.33) |
|  | Q4 (≥65%) | 35 / 288 | 12% | 1 (reference) | 1 (reference) |

*Logistic regression adjusted for age, sex, Charlson comorbidity index, APACHE Ⅱ score, and primary diagnosis category (sepsis, cerebrovascular diseases, cardiac diseases, cardiac arrest, respiratory diseases, gastrointestinal diseases, trauma, postoperative, and others). APACHE, Acute Physiology and Chronic Health Evaluation; HbA1c, glycosylated hemoglobin.
